# Delayed Diagnosis of Non-Bacterial Thrombotic Endocarditis in a Patient With Metastatic Lung Cancer

**DOI:** 10.7759/cureus.93398

**Published:** 2025-09-28

**Authors:** Efe Opone, Reza Aghamohammadzadeh

**Affiliations:** 1 Medicine, University Hospitals Sussex, Hove, GBR; 2 Cardiology, University Hospitals Sussex, Hove, GBR

**Keywords:** adenocarcinoma lung, cancer-associated thrombosis, embolic phenomena, endocarditis, lung cancer, non-infective thrombotic endocarditis, pericardial effusion (pe), thromboembolism and splenic infarction

## Abstract

Non-bacterial thrombotic endocarditis (NBTE) is characterised by sterile vegetations on cardiac valves and is most frequently associated with malignancy or chronic inflammatory disorders. Diagnosis is challenging, as clinical signs typically manifest after embolic complications.

We report the case of a 42-year-old man with a history of myocardial infarction and lacunar stroke earlier in the year, who presented with chronic cough, progressive dyspnoea, and fatigue. On admission, he was tachycardic and tachypneic, with a diastolic murmur and bibasal crackles. Laboratory tests revealed normocytic anaemia and thrombocytopenia. Computed tomography pulmonary angiogram (CTPA) excluded pulmonary embolism but demonstrated a large pericardial effusion, confirmed on transthoracic echocardiography, along with severe aortic regurgitation and a mobile echodensity on the aortic valve suspicious for vegetation. Pericardiocentesis drained 940 mL of haemoserous fluid, and cytology subsequently confirmed metastatic adenocarcinoma of probable pulmonary origin. Further imaging revealed bilateral renal and splenic infarcts. Serial blood cultures were negative. Given the combination of sterile vegetations, systemic embolisation, and advanced malignancy, a diagnosis of NBTE was made.

The patient was managed supportively, with surgical intervention deferred in view of disseminated malignancy and poor prognosis. His condition deteriorated rapidly, and he died several days after discharge.

This case illustrates the importance of considering NBTE in patients presenting with recurrent embolic events, particularly in the context of malignancy. Prompt recognition and initiation of anticoagulation may reduce the risk of further embolic complications, although prognosis remains poor in cancer-associated NBTE.

## Introduction

First described in 1888 by Zeigler and later named “marantic endocarditis” by Libman in 1923, non-bacterial thrombotic endocarditis (NBTE) is a form of non-infective thrombotic endocarditis characterised by sterile fibrin-platelet vegetations on cardiac valve leaflets [1]. It most commonly affects the mitral and aortic valves and is strongly associated with malignancy, particularly mucin-producing adenocarcinomas, and chronic inflammatory conditions such as systemic lupus erythematosus and antiphospholipid syndrome [2].

Although uncommon, NBTE is clinically important because it frequently presents with systemic embolic events rather than cardiac symptoms, often delaying diagnosis [3]. In a contemporary Cleveland Clinic registry spanning 20 years, stroke accounted for ≈60% of presentations, while acute coronary syndrome represented 7% [4]. A recent systematic review and meta-analysis of 450 patients reported that approximately 70% had embolic phenomena and that malignancy was associated with a sixfold increase in embolic risk and a significantly higher in-hospital mortality [5].

Antemortem diagnosis remains challenging despite advances in echocardiographic imaging, and there are currently no formal guidelines for investigation or management [6].

We report the case of a 42-year-old man with recurrent embolic events, pericardial effusion, and valvular regurgitation, ultimately diagnosed with NBTE secondary to metastatic lung adenocarcinoma. This case highlights the importance of maintaining a high index of suspicion for NBTE in patients with unexplained systemic embolisation and the value of multimodal imaging in establishing the diagnosis.

## Case presentation

A 42-year-old man presented with chronic cough, progressive shortness of breath, and fatigue. He denied chest pain, weight loss, rashes, joint pain, or gastrointestinal/genitourinary symptoms but reported occasional night sweats. His history was significant for two vascular events earlier in the year, a lacunar stroke and a non-ST elevation myocardial infarction, raising concern for an underlying prothrombotic state. He had a family history of ischaemic heart disease and reported cannabis use and alcohol excess.

On admission, he was tachypneic with a respiratory rate of 24 breaths per minute, oxygen saturation 99% on air, blood pressure 131/90 mmHg, heart rate 127 beats/min, and temperature 35.6°C. He appeared pale, with digital clubbing. Cardiovascular examination revealed a grade 3 diastolic murmur along the left sternal border, and bilateral crackles were heard on chest auscultation. Laboratory investigations demonstrated normocytic anaemia, thrombocytopenia, and elevated urea (Table 1).

**Table 1:** Laboratory parameters on admission and day two Hb: Haemoglobin, WCC: White Cell Count, MCV: Mean Corpuscular Volume, INR: International Normalised Ratio, eGFR: Estimated Glomerular Filtration Rate, CRP: C-Reactive Protein, C3: Complement Component 3, C4: Complement Component 4, IgG Anticardiolipin Ab: Immunoglobulin G Anticardiolipin Antibody, IgM Anticardiolipin Ab: Immunoglobulin M Anticardiolipin Antibody, IgG B-2-glycoprotein-1 Ab: Immunoglobulin G Beta-2 Glycoprotein 1 Antibody, IgM B-2 glycoprotein-1 Ab: Immunoglobulin M Beta-2 Glycoprotein 1 Antibody, CTD screen: Connective Tissue Disease Screen, MPO Ab: Myeloperoxidase Antibody, PR3 Ab: Proteinase 3 Antibody.

	Day 1	Day 2	Reference range	Units
Hb	56	64	130-180	g/L
WCC	10.4	9.9	3.6 - 11.0	x10^9/L
Platelets	76	70	140 - 400	x10^9/L
MCV	94	95	80 - 100	fL
Neutrophils	8	6.4	1.8 - 7.5	x10^9/L
INR	1.5	1.3	0.8-1.2	-
Fibrinogen	0.9	-	1.5-4.5	g/L
Sodium	136	134	133 - 146	mmol/L
Potassium	4.4	4.1	3.5-5	mmol/L
eGFR	>90	>90	>90	mL/min/1.73m²
CRP	8	45	5	mg/L
Troponin I	306	-	0-34	ng/L
D dimer	3048	-	0-229	Ng/mL
C3	1.5	-	0.8-1.9	g/L
C4	0.36	-	0.2-0.5	g/L
IgG Anticardiolipin Ab	1.5	-	0-10	GPL U/mL
IgM Anticardiolipin Ab	3.1	-	0-10	GPL U/mL
IgG B-2-glycoprotein-1 Ab	<0.8	-	0-10	GPL U/mL
IgM B-2 glycoprotein-1 Ab	8.1	-	0-10	GPL U/mL
CTD screen	0.1	-	0-1	-
MPO Ab	<0.2	-	0-5	IU/mL
PR3 Ab	<0.6	-	0-3	IU/mL

**Figure 1:**
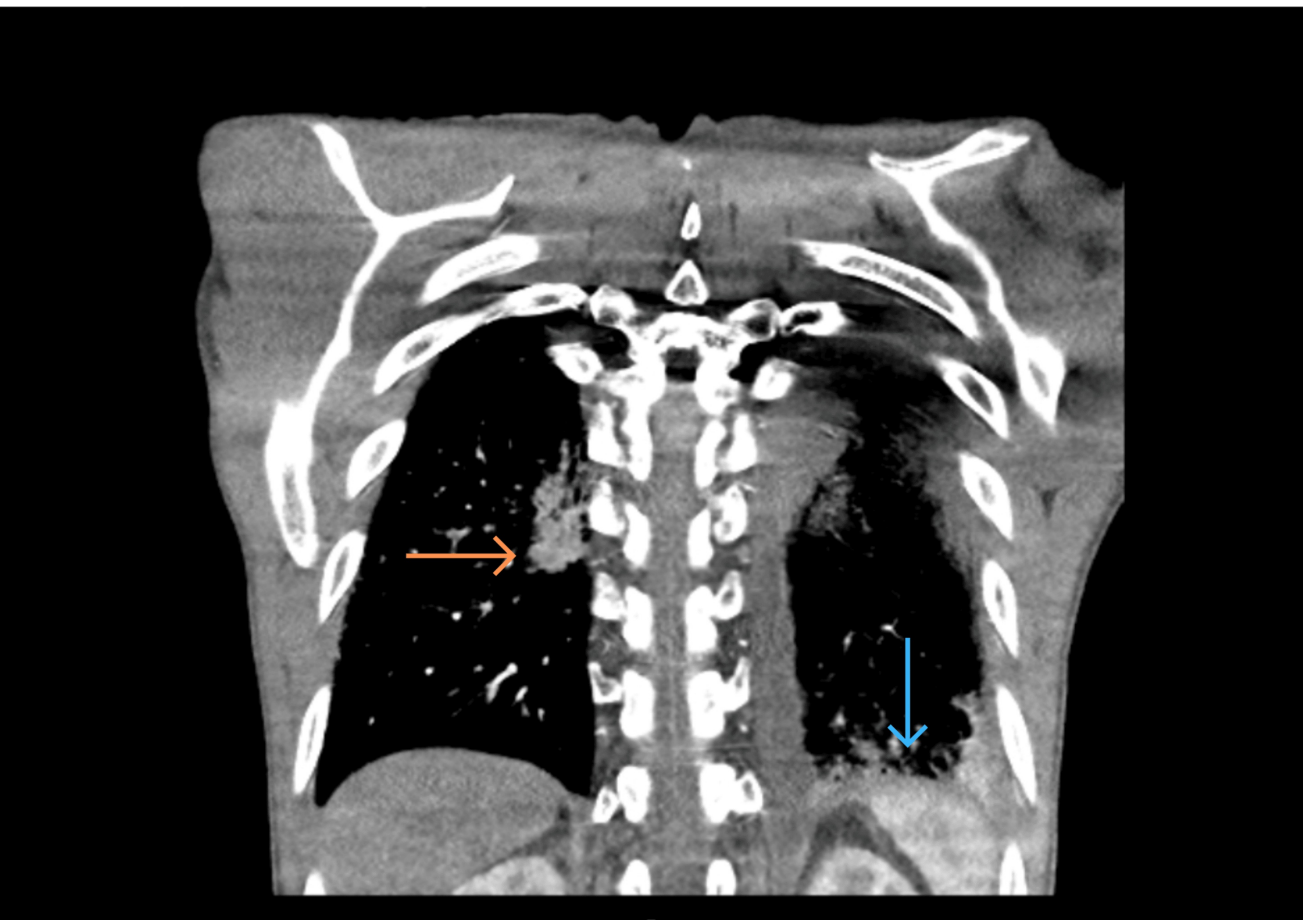
CTPA coronal view – right subpleural nodular consolidation (orange arrow) and consolidative changes in the left lower lobe (blue arrow) CTPA: Computed tomography pulmonary angiogram

**Figure 2:**
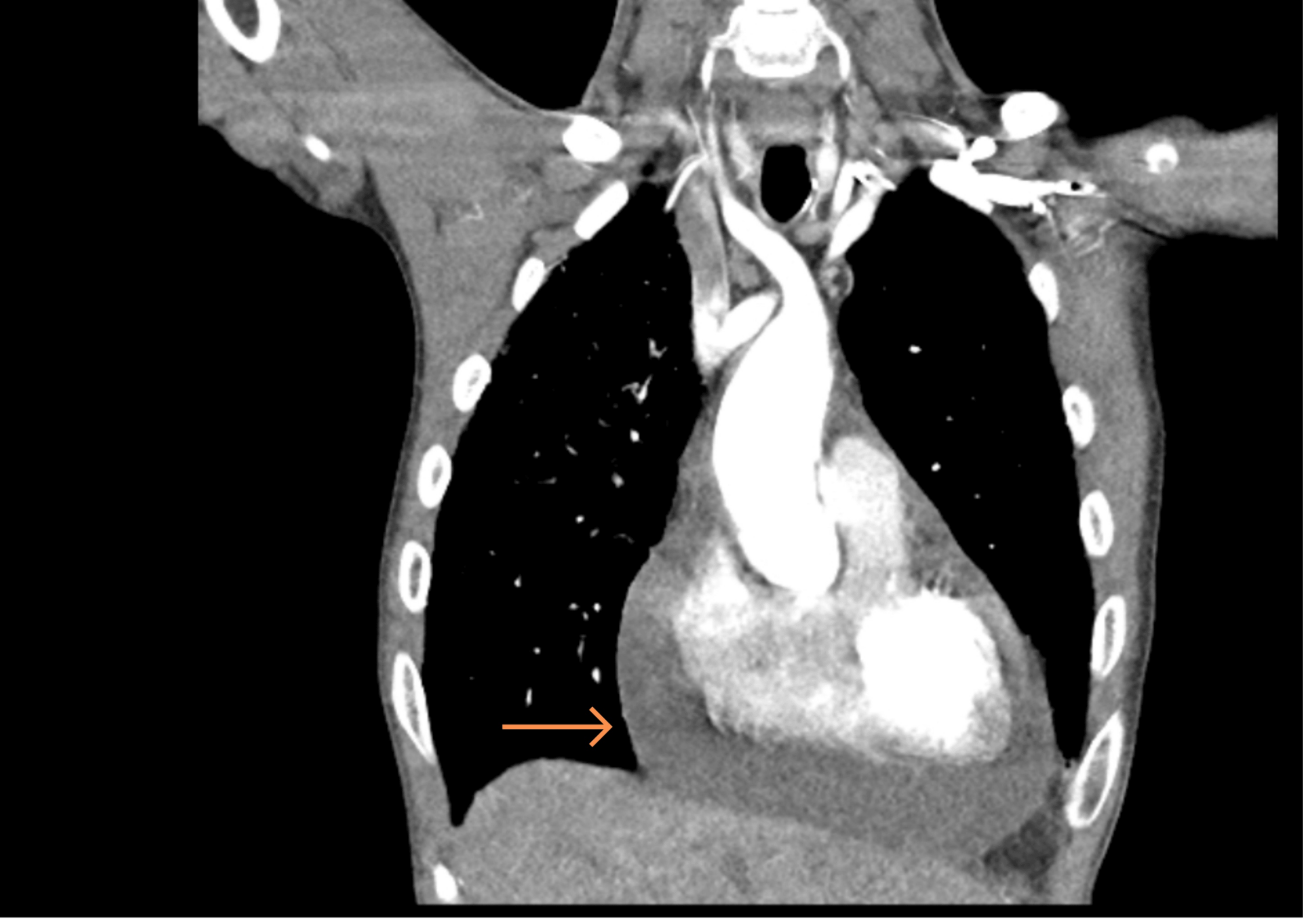
CTPA coronal view – moderate to gross volume pericardial effusion (orange arrow) CTPA: Computed tomography pulmonary angiogram

**Figure 3:**
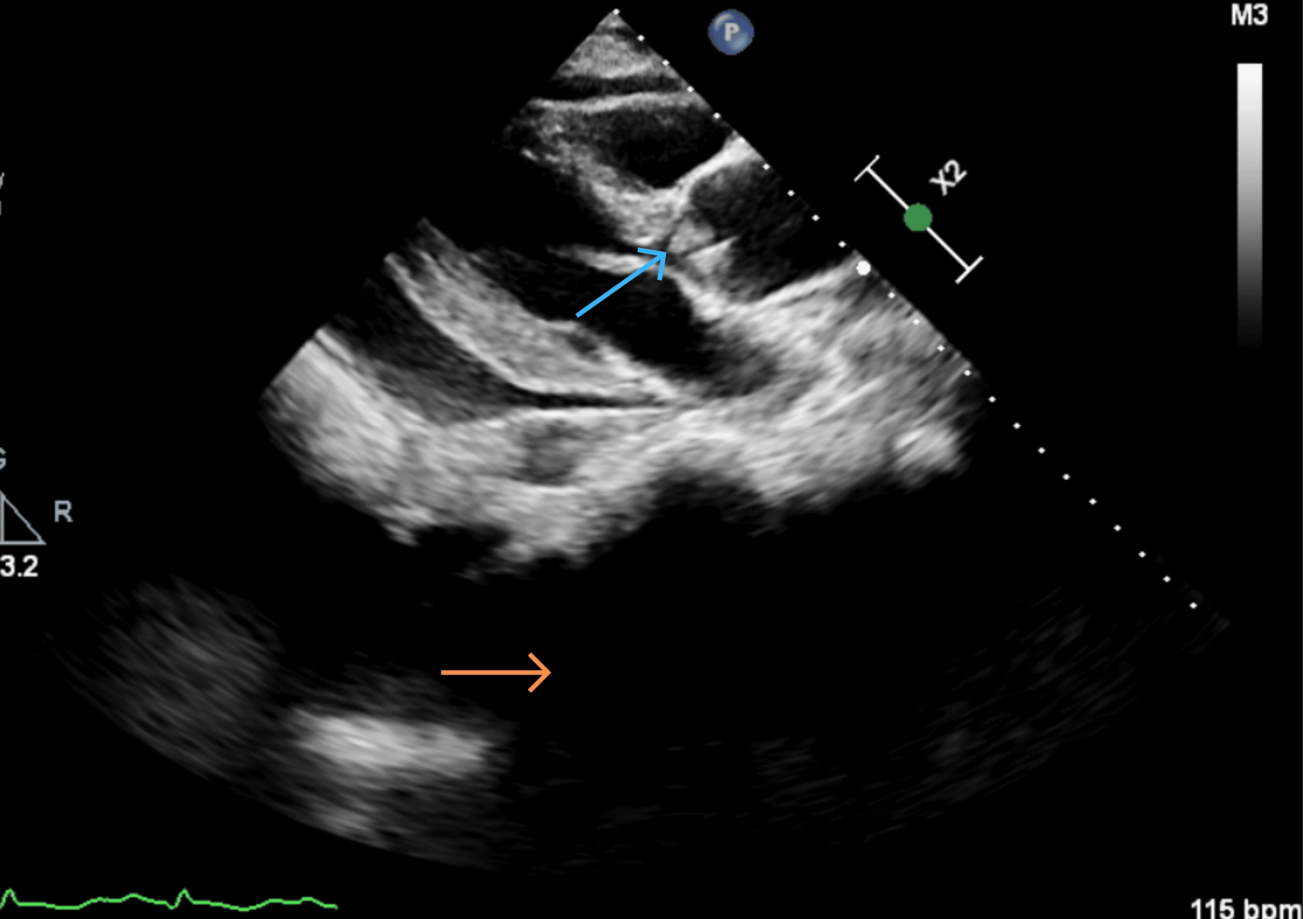
TTE PLAX view – pericardial effusion (orange arrow) and thick aortic valve (blue arrow) TTE: Transthoracic echocardiogram, PLAX: Parasternal long axis

**Figure 4:**
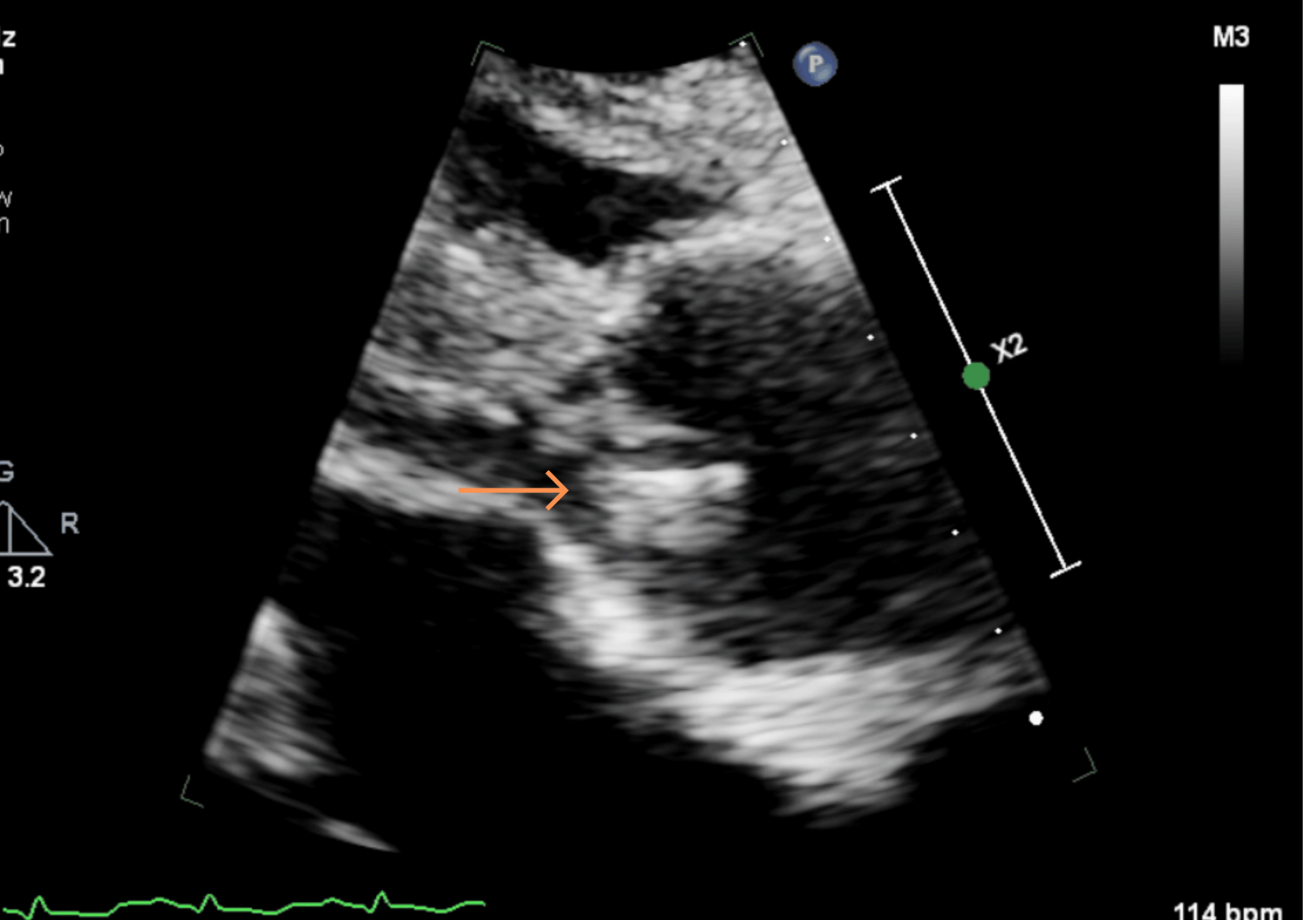
TTE PLAX view – zoom in on the aortic valve (orange arrow) TTE: Transthoracic echocardiogram, PLAX: Parasternal long axis

**Figure 5:**
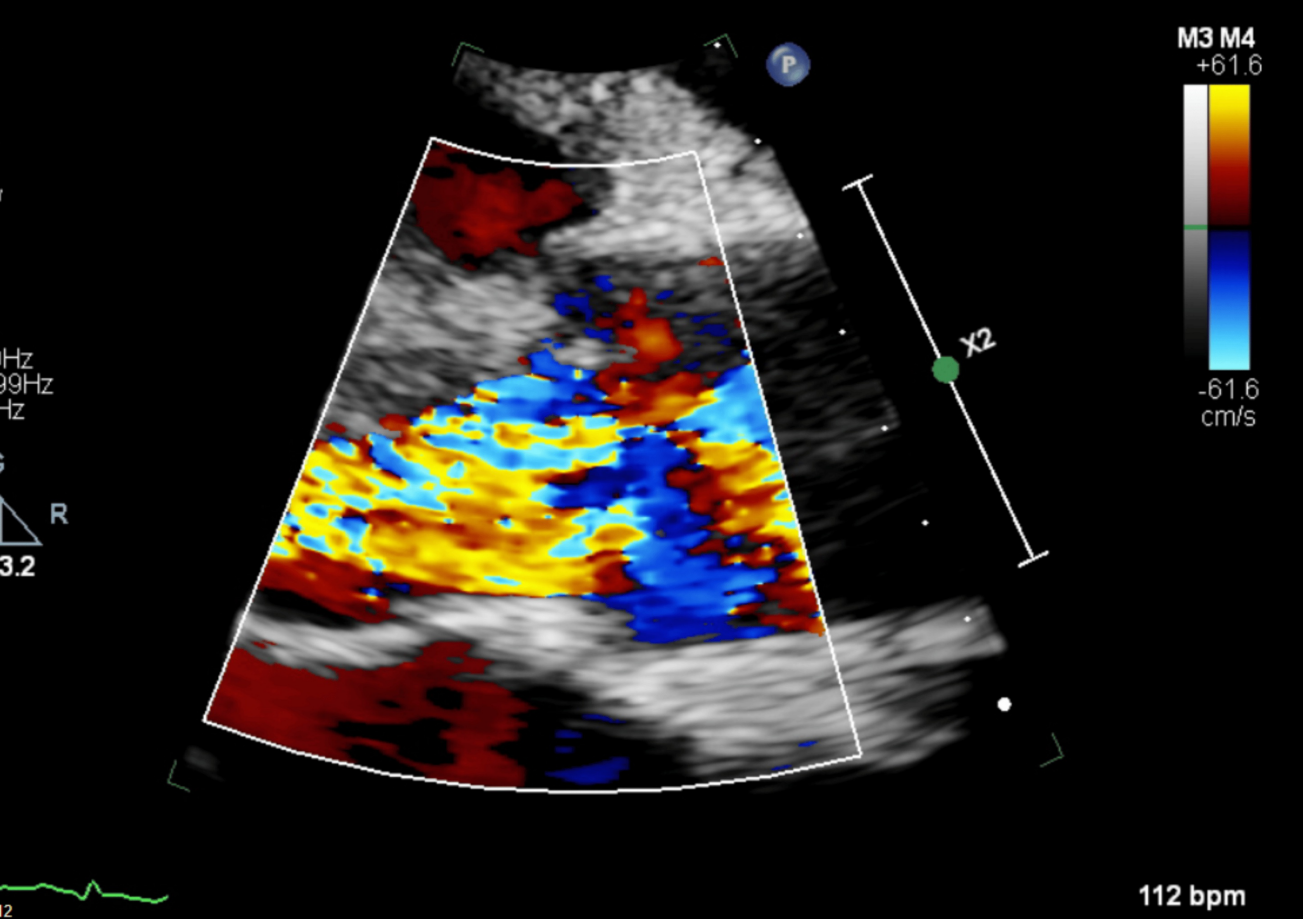
TTE PLAX view - zoom in on the aortic valve with colour flow mapping showing the aortic regurgitation TTE: Transthoracic echocardiogram, PLAX: Parasternal long axis

**Figure 6:**
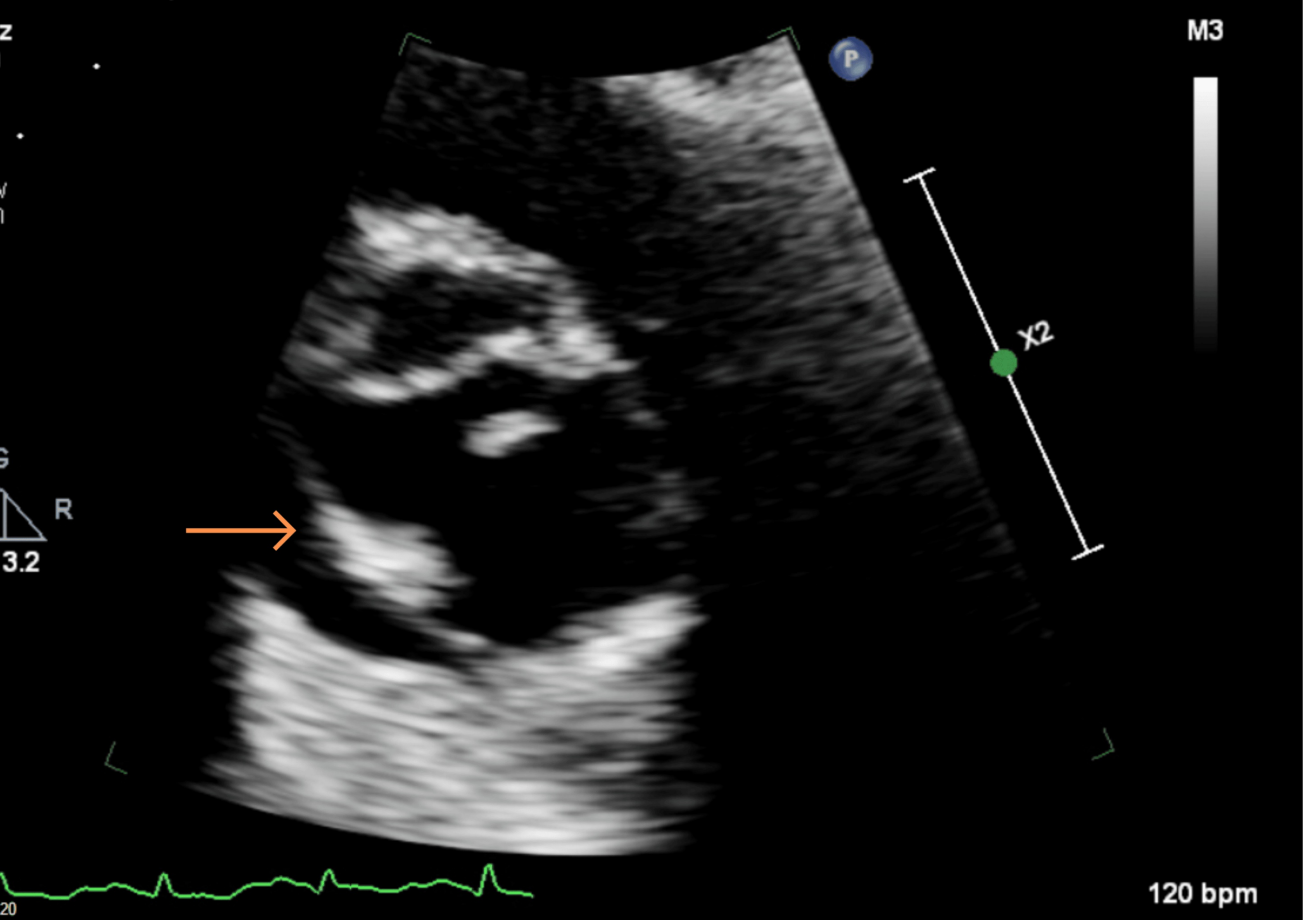
TTE PSAX view - aortic valve and the possible vegetation structure (orange arrow) TTE: Transthoracic echocardiogram, PSAX: Parasternal short axis

**Figure 7:**
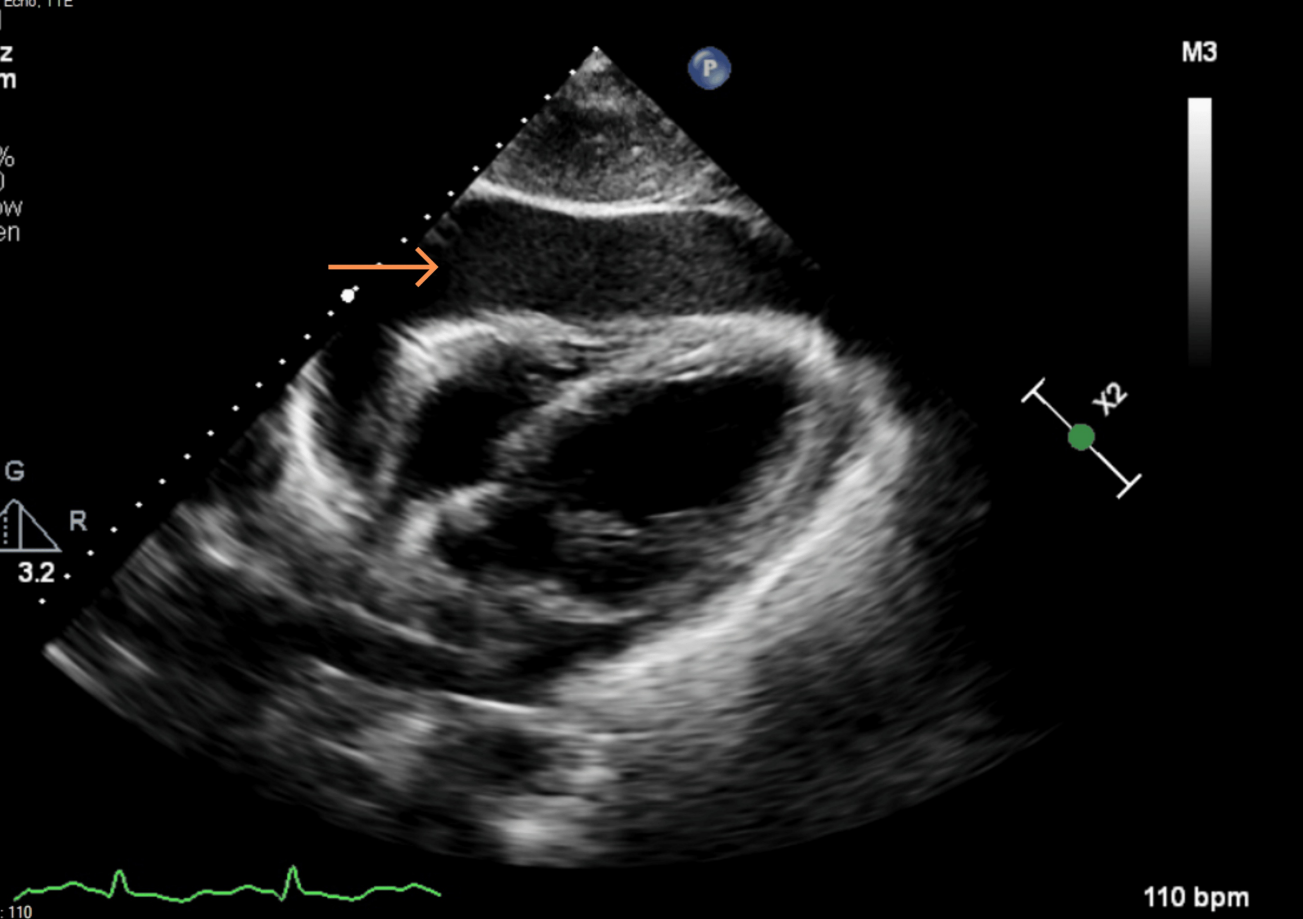
TTE subcostal window - pericardial effusion (orange arrow) TTE: Transthoracic echocardiogram

Given the anaemia, an oesophagogastroduodenoscopy was performed but revealed no evidence of upper gastrointestinal bleeding. In view of his marked tachypnoea, persistent sinus tachycardia, and previous thromboembolic events, a computed tomography pulmonary angiogram (CTPA) was requested to exclude pulmonary embolism as a potentially reversible cause of his acute deterioration. The study demonstrated no filling defects within the pulmonary arteries but revealed a moderate-to-large pericardial effusion, a small left pleural effusion, and interstitial thickening in the right lung lobes (Figures 1, 2). Transthoracic echocardiography confirmed a large pericardial effusion (2.8 cm anteriorly), severe aortic regurgitation, and a mobile echodensity attached to the left coronary cusp of the aortic valve, suspicious for vegetation (Figures 3-7).

Pericardiocentesis drained 940 mL of haemoserous fluid for cytology and culture. Additional CT imaging demonstrated peripheral wedge-shaped infarcts in both kidneys and two large splenic infarcts, consistent with systemic embolisation. Serial blood cultures remained negative, and serology for Brucella and Coxiella burnetii was also negative.

The case was discussed at a multidisciplinary cardiology meeting, and the patient was considered for aortic valve replacement pending transoesophageal echocardiography and preoperative assessment. Despite empirical antibiotic therapy, follow-up imaging demonstrated worsening interstitial lung changes and new infrarenal aortic stenosis.

Cytological analysis of the pericardial fluid subsequently confirmed metastatic adenocarcinoma of probable pulmonary origin. In view of the diagnosis of advanced malignancy, sterile vegetations on echocardiography, persistently negative blood cultures, and recurrent systemic emboli, a diagnosis of non-bacterial thrombotic endocarditis (NBTE) was favoured. In light of the poor overall prognosis, surgical intervention was deferred, and a palliative approach was adopted. The patient’s condition deteriorated rapidly, and he died several days after discharge.

## Discussion

This case highlights the diagnostic challenge of NBTE when the presenting feature is recurrent systemic embolisation rather than cardiac symptoms. NBTE is an uncommon but clinically significant entity that most often affects the mitral and aortic valves [2,4-9]. Diagnosis is frequently delayed, and embolic events, particularly stroke, are the most common presenting manifestation [7]. A Cleveland Clinic registry reported that stroke accounted for approximately 60% of initial presentations, with acute coronary syndrome occurring in 7% [4].

The most comprehensive epidemiological summary to date comes from a recent systematic review and meta-analysis of 416 reports comprising 450 patients [5]. The median age at diagnosis was 48 years, closely mirroring the age of our patient. The female-to-male ratio was approximately 2:1. Embolic phenomena were observed in around 70% of cases, with stroke being the most frequent manifestation. Malignancy was the leading associated condition and was strongly linked to an increased risk of embolic complications (adjusted odds ratio ≈ 6.4 compared with non-malignant causes). In-hospital mortality was high overall (36%), rising to more than 50% in cancer-associated NBTE, again consistent with the poor prognosis seen in our case, where the patient deteriorated rapidly despite supportive management.

Rogers et al. conducted a seminal clinical and pathological study of 115 patients with cancer and NBTE identified at autopsy [8]. Forty-two of these patients had cerebral infarcts attributable to NBTE rather than to other vascular pathology. Carcinoma of the lung was the most common malignancy, accounting for 33% of cases, followed by tumours of the gastrointestinal tract and haematopoietic system, findings which closely align with the primary lung adenocarcinoma identified in our patient. Importantly, Rogers et al. also investigated the effects of anticoagulation and demonstrated a reduction in the number and size of cerebral infarcts in anticoagulated patients, supporting the use of anticoagulation as a key component of management where not contraindicated.

The pathophysiology of NBTE is rooted in a hypercoagulable state. Malignancy contributes through tumour cell expression of procoagulant factors (e.g., tissue factor), vascular compression by tumour masses, and a systemic pro-inflammatory response [2]. Chronic inflammatory conditions, including systemic lupus erythematosus, rheumatoid arthritis, and antiphospholipid syndrome, are also recognised triggers; in one surgical series, 60% of patients with NBTE had an immune-mediated disorder [9].

Historically, NBTE was diagnosed almost exclusively at post-mortem, but advances in cardiac imaging, particularly transoesophageal echocardiography, have facilitated antemortem recognition [6]. Despite this, diagnosis remains challenging, and there is no widely accepted guidance for investigation or treatment. Several authors have recently proposed diagnostic algorithms, but maintaining a high index of suspicion remains crucial. NBTE should be strongly considered in patients presenting with unexplained systemic embolisation, especially in the setting of malignancy or inflammatory disease. Our case exemplifies these principles: the combination of recurrent embolic events, sterile vegetations on echocardiography, negative blood cultures, and the subsequent discovery of metastatic adenocarcinoma was highly suggestive of NBTE and directed management towards palliative care rather than surgical intervention.

## Conclusions

Nonbacterial thrombotic endocarditis (NBTE) remains a challenging and frequently under-recognised diagnosis because of its non-specific presentation and tendency to manifest only after systemic embolisation. Our case is noteworthy for several reasons: it involved a young man with no known cancer diagnosis at presentation, whose first two clinical manifestations were myocardial infarction and lacunar stroke, both unusual sentinel events for NBTE. In addition, the coexistence of severe aortic regurgitation, a large haemorrhagic pericardial effusion, and sterile vegetations is rarely reported together in the context of NBTE.

This case demonstrates that malignancy can present with multisystem thromboembolic disease mimicking infective endocarditis or autoimmune disorders and highlights the need for clinicians to maintain a high index of suspicion for NBTE when faced with unexplained embolic events, valve abnormalities, or recurrent vascular occlusion, even in the absence of confirmed infection. Early recognition, prompt echocardiography, and consideration of anticoagulation may reduce further embolic complications. Despite advances in imaging, there remains a paucity of formal diagnostic and management guidelines for NBTE, particularly in cancer-associated cases. Reporting rare presentations such as this contributes to a growing body of evidence that may ultimately inform future algorithms for earlier detection and treatment.
